# Worked to the bone: antibody-based conditioning as the future of transplant biology

**DOI:** 10.1186/s13045-022-01284-6

**Published:** 2022-05-19

**Authors:** James M. Griffin, Fiona M. Healy, Lekh N. Dahal, Yngvar Floisand, John F. Woolley

**Affiliations:** 1 Department of Pharmacology and Therapeutics, University of Liverpool, Liverpool, UK; 2 Department of Molecular and Clinical Cancer Medicine, University of Liverpool, Liverpool, UK; 3 The Clatterbridge Cancer Centre NHS Foundation Trust, Liverpool, UK

**Keywords:** Conditioning, Stem cell transplant, Graft-versus-host disease, Graft versus leukaemia, Antibody–drug conjugate, Monoclonal antibody, Immunotherapy

## Abstract

Conditioning of the bone marrow prior to haematopoietic stem cell transplant is essential in eradicating the primary cause of disease, facilitating donor cell engraftment and avoiding transplant rejection via immunosuppression. Standard conditioning regimens, typically comprising chemotherapy and/or radiotherapy, have proven successful in bone marrow clearance but are also associated with severe toxicities and high incidence of treatment-related mortality. Antibody-based conditioning is a developing field which, thus far, has largely shown an improved toxicity profile in experimental models and improved transplant outcomes, compared to traditional conditioning. Most antibody-based conditioning therapies involve monoclonal/naked antibodies, such as alemtuzumab for graft-versus-host disease prophylaxis and rituximab for Epstein–Barr virus prophylaxis, which are both in Phase II trials for inclusion in conditioning regimens. Nevertheless, alternative immune-based therapies, including antibody–drug conjugates, radio-labelled antibodies and CAR-T cells, are showing promise in a conditioning setting. Here, we analyse the current status of antibody-based drugs in pre-transplant conditioning regimens and assess their potential in the future of transplant biology.

## Introduction

Haematopoietic stem cell transplantation (HCT) is a potentially curative modality of treatment for patients with a variety of genetic disorders or malignancies, such as leukaemia, lymphoma and anaemia [1]. The importance of HCT is underlined by the fact that over 1,300,000 procedures were performed in World Health Organisation (WHO) member countries between 2006 and 2014 [2]. Following HCT, donor stem cells reconstitute the host haematopoietic system with healthy mature blood cells. Successful HCT is reliant on many factors, including human leukocyte antigen (HLA) compatibility, sustained engraftment, avoidance of serious graft-versus-host disease (GvHD) and effective pre-transplant conditioning. This review will focus on the conditioning regimen, which serves the purposes of disease eradication, bone marrow (BM) depletion to create space for donor cells, and to reduce immune-driven rejection [3].

HCT is not without risks. Early and late post-transplant (< 100/ > 100 days) mortality is common due to the significant toxicity placed upon the body by the conditioning process [1]. Conditioning regimens are classically divided into two groups: myeloablative conditioning (MAC) and reduced intensity conditioning (RIC), which are categorised based on the extent of BM haematopoietic ablation and the requirement for stem cell (SC) support [4], although new methods, such as the transplant conditioning intensity score, are being developed to address limitations in the conventional classification system [5]. MAC regimens irreversibly ablate marrow haematopoiesis, therefore requiring SC support, whereas RIC regimens do not require support, as variable and reversible cytopenia is observed [4].

Conditioning regimens classically involve the same backbone—radiotherapy, often total body irradiation (TBI) and chemotherapy such as cyclophosphamide, treosulfan, melphalan, thiotepa, busulfan or fludarabine [6]. TBI is associated with potentially fatal gastrointestinal, hepatic and pulmonary toxicities. Chemotherapy agents cause varying toxicities, including pulmonary, hepatic and nephrotoxicity. Both TBI and chemotherapy can cause sinusoidal obstructive syndrome (SOS) and secondary malignancies [6]. This toxicity often restricts which patients are eligible for transplant due to their fitness, such as older patients or those with co-morbidities.

**Fig. 1:**
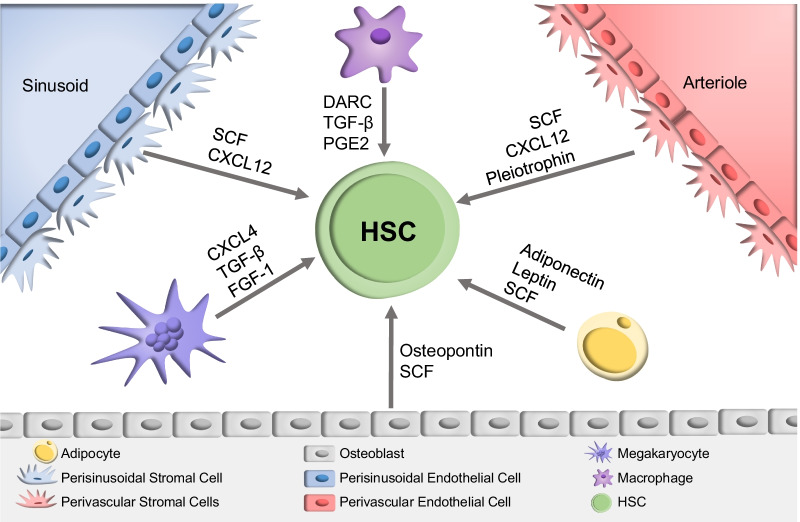
Cell interactions in the HSC niche. Multiple cell types make up the haematopoietic stem cell (HSC) niche, which interacts to influence critical factors such as HSC homing, maintenance, survival and proliferation, through the release of cytokines. Macrophages influence HSC maintenance and quiescence through the actions of DARC, PGE2 and TGF-β, respectively. Arteriole and sinusoidal stromal cells promote HSC maintenance and self-renewal via SCF, CXCL12, as well as expansion through pleiotrophin. Adipocytes have varied roles, including the impairment of HSC proliferation through adiponectin, whereas leptin releases promote proliferation and expansion. The production of osteopontin by osteoblasts induces HSC quiescence, as does CXCL4 and TGF-β produced by megakaryocytes. On the other hand, FGF-1 release from megakaryocytes promotes HSC proliferation and recovery, as well as niche remodelling after radiation. [7–9, 133]. *SCF*: stem cell factor, *PGE2*: prostaglandin E2, *CXCL12*: C-X-C motif chemokine ligand 12, *CXCL4*: C-X-C motif ligand 4, *TGF-B*: transforming growth factor beta, *FGF-1*: fibroblast growth factor, *DARC*: Duffy antigen receptor for cytokines

Despite toxicities, conditioning is essential for effective HCT for many reasons. For instance, conditioning creates space in the BM microenvironment and HSC niches, both highly complex structures [7, 8]. Spatial organisation of HSCs in highly complex perivascular and endosteal niches is key, as many different cell types provide molecular cues that promote HSC self-renewal, BM retention and proliferation (Fig. 1). Conditioning empties these niches, providing space for donor HSCs to enter and receive the signals necessary for them to reconstitute the haematopoietic system [7–9]. Space in the BM microenvironment is also necessary for granulocyte–macrophage progenitors (GMPs), which become tightly packed and surrounded by mature cells that release cytokines that promote expansion during times of regenerative stress, such as after HCT [10, 11]. Lastly, it has been shown that conditioning stimulates the BM niche to produce factors that promote engraftment, such as pleiotrophin (PTN) [12]. Conditioning also induces immunosuppression to abrogate the risk of graft rejection and GvHD, using agents such as anti-thymocyte globulin (ATG), cyclosporine, tacrolimus, sirolimus and mycophenolate mofetil [13, 14].

**Fig. 2:**
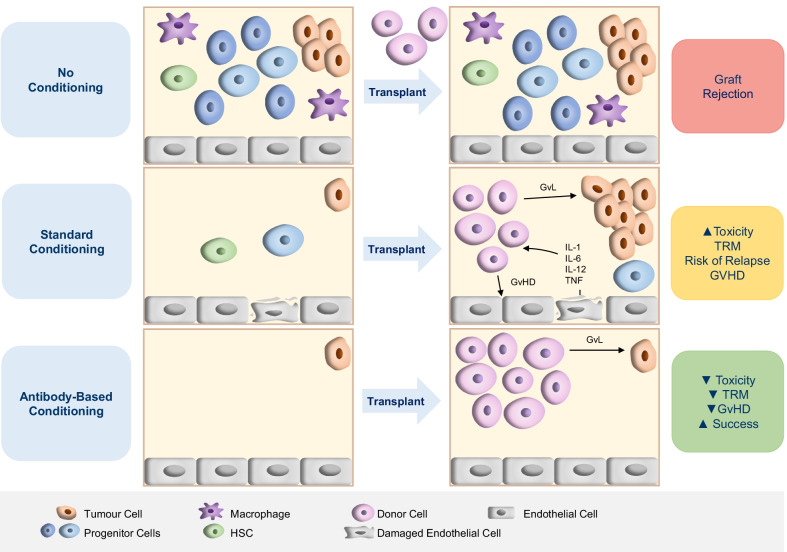
Potential outcomes of different conditioning regimens in regards to transplant success. Top row without conditioning, the bone marrow (BM) is not depleted. After transplant, the lack of space, immunosuppression and disease eradication mean the donor cells do not engraft, leading to graft rejection and transplant failure. Middle row using standard conditioning: the BM is depleted, but the toxicity of the regimen leads to tissue damage. Reduced intensity conditioning (RIC) causes incomplete BM depletion. After transplant, the graft cells have space to engraft; however, the tissue damage leads to the release of inflammatory cytokines, which can induce graft-versus-host disease (GvHD). The donor cells can also mount a graft-versus-leukaemia (GvL) response against residual malignant cells. Using RIC, there is a risk of disease relapse caused by the outgrowth of residual malignant cells. The toxicity of conditioning is associated with transplant-related mortality (TRM). Bottom row antibody-based conditioning can lead to effective and targeted clearance of the BM niche, eliminating HSCs and progenitor cells while sparing host tissue by specific targeting of expressed CD markers. This can cause minimal toxicity and therefore reduce GvHD and TRM. For example, antibodies such as alemtuzumab and vedolizumab have been shown to reduce GvHD incidence, while radio-labelled antibodies such as ibritumomab tiuxetan have shown favourable toxicity profiles vs traditional TBI. Many pre-clinical ADCs have reported extremely impressive levels of BM clearance and engraftment after HCT in mouse models, which would positively impact transplant success if translated to human studies. It should be noted that not all antibody-based therapies provide such benefits, such as early studies associating gemtuzumab ozogamicin with increased toxicity. After transplant, the donor cells can successfully engraft due to effective clearance of the niche. Furthermore, the donor cells eliminate residual malignant cells by the graft-versus-leukaemia (GvL) effect

**Fig. 3:**
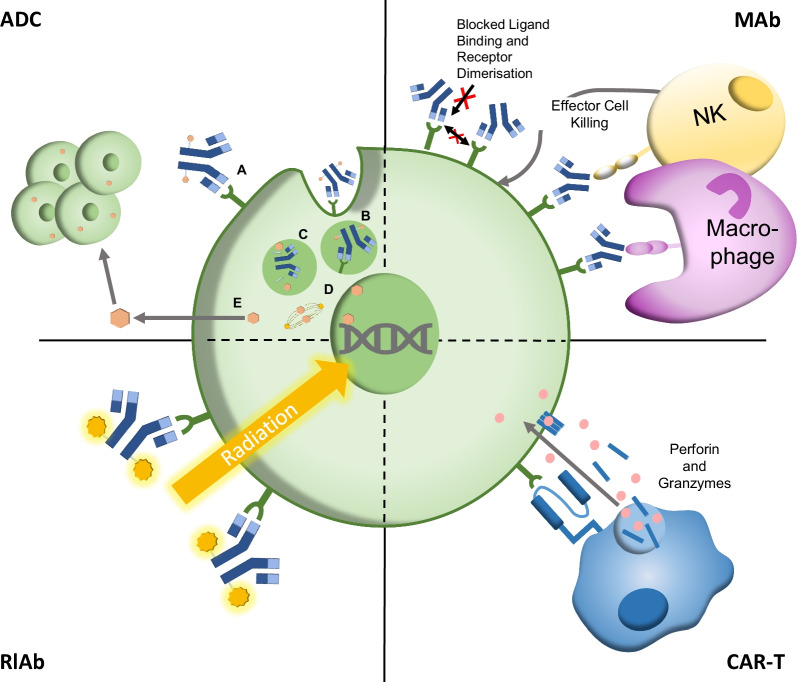
Molecular mechanism of antibody-based bone marrow conditioning. Antibody–drug conjugates (ADC, Top Left) **A** An ADC binds to its antigen receptor, then **B** the ADC–receptor complex is endocytosed into the cytosol in an endosome before **C** being trafficked to a lysosome, where the linker is cleaved, releasing the toxic payload into the cytosol **D** where it exerts a cytotoxic effect, typically by microtubule inhibition or DNA intercalation. **E** The payload can exit the cell and enter neighbouring cells, killing them (bystander effect). Monoclonal antibodies (MAb, Top Right) binding to receptors blocks ligand binding and receptor dimerisation, blocking cell survival signals. Effector cells such as macrophages and NK cells are recruited by recognising Fc region of antibody. CAR-T-engineered receptor of CAR-T recognises antigen and exerts cytotoxic effects, such as the release of perforin and granzymes to cause cell lysis. RlAb radio-labelled antibody binds to antigen and payload causes radiation-induced cell damage

Conditioning-associated toxicities are largely due to off-target effects, whereby the agents damage cells not found within the BM [6]. To this end, more specific conditioning agents could limit off-target toxicities and facilitate a higher targeted dose to the marrow, increasing BM depletion and transplant success while reducing the rate of relapse (Fig. 2). Therefore, antibody- or immunotherapy-based conditioning regimens may be the future for HCT. Antibody therapies eliminate specific cell types by targeting antigens on the cell surface, typically cluster of differentiation (CD) markers that allow the identification of certain cell types, with each format eliminating cells using different mechanisms (Fig. 3).

Currently, 133 antibody therapeutics of various formats, including unconjugated, antibody–drug conjugates (ADCs) and radio-labelled antibodies have been approved or are under review by the FDA, for a range of diseases (Table 1), [15]. In 2020, 13 of the 53 FDA-approved drugs were biologics, 10 of those being monoclonal antibodies and 2 ADCs [16]. Furthermore, many other immunotherapy-based compounds are at varying stages of development for use in conditioning (Table 2). This review will summarise recent advancements and trials of antibody-based agents in pre-HCT BM conditioning.

**Table 1:** Antibody-based therapies approved or under review for haematological or transplant-related disorders

International non-proprietary name	Brand name	Target	Format	Conjugated/unconjugated	Indication first approved or reviewed	First US approval year
Muromonab-CD3	Orthoclone Okt3	CD3	Full-length antibody	Unconjugated	Reversal of kidney transplant rejection	1986
Abciximab	Reopro	GPIIb/IIIa	Fab	Unconjugated	Prevention of blood clots in angioplasty	1994
Basiliximab	Simulect	IL-2R	Full-length antibody	Unconjugated	Prevention of kidney transplant rejection	1998
Rituximab	MabThera, Rituxan	CD20	Full-length antibody	Unconjugated	NHL	1997
Trastuzumab	Herceptin	HER2	Full-length antibody	Unconjugated	Breast cancer	1998
Gemtuzumab ozogamicin	Mylotarg	CD33	Full-length antibody	ADC	AML	2017; 2000
Alemtuzumab	Lemtrada; MabCampath, Campath-1H	CD52	Full-length antibody	Unconjugated	Multiple sclerosis; CML	2014; 2001
Ibritumomab tiuxetan	Zevalin	CD20	Full-length antibody	Radio-immunotherapeutic	NHL	2002
Tositumomab-I131	Bexxar	CD20	Full-length antibody	Radio-immunotherapeutic	NHL	2003
Eculizumab	Soliris	C5	Full-length antibody	Unconjugated	Paroxysmal nocturnal haemoglobinuria	2007
Ofatumumab	Arzerra	CD20	Full-length antibody	Unconjugated	CLL	2009
Brentuximab vedotin	Adcetris	CD30	Full-length antibody	ADC	HL, sALCL	2011
Obinutuzumab	Gazyva, Gazyvaro	CD20	Full-length antibody	Unconjugated	CLL	2013
Idarucizumab	Praxbind	Dabigatran	Fab	Unconjugated	Reversal of dabigatran-induced anticoagulation	2015
Blinatumomab	Blincyto	CD19, CD3	Tandem scFv	Unconjugated	ALL	2014
Daratumumab	Darzalex	CD38	Full-length antibody	Unconjugated	MM	2015
Elotuzumab	Empliciti	SLAMF7	Full-length antibody	Unconjugated	MM	2015
Inotuzumab ozogamicin	BESPONSA	CD22	Full-length antibody	ADC	ALL	2017
Emicizumab	Hemlibra	Factor Ixa, X	Full-length antibody	Unconjugated	Haemophilia A	2017
Ravulizumab	Ultomiris	C5	Full-length antibody	Unconjugated	Paroxysmal nocturnal haemoglobinuria	2018
Moxetumomab pasudotox	Lumoxiti	CD22	dsFv immunotoxin	Immunotoxin	Hairy cell leukaemia	2018
Crizanlizumab	Adakveo	CD62	Full-length antibody	Unconjugated	Sickle cell disease	2019
Polatuzumab vedotin	Polivy	CD79b	Full-length antibody	ADC	DLBCL	2019
Isatuximab	Sarclisa	CD38	Full-length antibody	Unconjugated	MM	2020
Belantamab mafodotin	BLENREP	BCMA	Full-length antibody	ADC	MM	2020
Tafasitamab	Monjuvi, Minjuvi	CD19	Full-length antibody	Unconjugated	DLBCL	2020
Loncastuximab tesirine	Zynlonta	CD19	Full-length antibody	ADC	DLBCL	2021
Narsoplimab	(Pending)	MASP-2	Full-length antibody	Unconjugated	HSCT-Associated thrombotic microangiopathies	In review
Ublituximab	(Pending)	CD20	Full-length antibody	Unconjugated	CLL	In review
Teclistamab	(Pending)	BCMA, CD3	Full-length antibody	Unconjugated	MM	In review

Information from the antibody society [15]

*AML*: acute myeloid leukaemia, *ALL*: acute lymphoblastic leukaemia, *CML*: chronic myeloid leukaemia, *CLL*: chronic lymphocytic leukaemia, *DLBCL*: diffuse large B cell lymphoma, *HL*: Hodgkin lymphoma, *MM*: multiple myeloma, *NHL*: non-Hodgkin lymphoma, *sALCL*: systemic anaplastic large-cell lymphoma

**Table 2:** Antibodies in development for pre-HCT bone marrow conditioning

Antibody-based therapy name	Target and format	Phase of trial(s)	Underlying disease	Sample number	Key outcomes	Ref
Vedolizumab	α4β7 integrin MAb	Phase 1b	AML, ALL, MDS	24: 3 Low Dose (LD), 21 High Dose (HD)	TTE: LD = 22, HD = 14 aGvHD 2–4: LD = 0%, HD = 19% 1 yr OS: LD = 66.6%, HD = 84.7%	[40]
Vedolizumab	α4β7 integrin MAb	Phase 3	Haem malignancy, myeloproliferative disorder	343	Ongoing	[41]
JSP191	CD117 MAb	Phase 1/2	AML, MDS, SCID, FA	40, 40, 12 (Estimated)	Ongoing	[46–49]
FSI-174 + Magrolimab	CD117MAb + CD47 MAb	Rhesus Macaques	None	Undisclosed	Significant depletion of HSCs	[43]
ACK-2 + anti-CD47 MAb	Anti-Mouse CD117 Mab	Mice	None	–	> 99% HSC depletion	[50]
Six-antibody cocktail	Anti-CD4, CD8, CD40L, CD47, CD117, CD122	Mice	None	5	52% Donor granulocyte chimerism at 8 weeks	[51]
CD45-SAP	CD45 ADC	Mice	None	5	99% Host HSC depletion 4mo 90% HSC donor chimerism Reduced toxicity versus TBI	[53]
CD45-SAP	CD45 ADC	Mice	SCID	–	91.7–95/2% Host HSC depletion 32.04–100% Donor HSC chimerism	[55]
CD117-SAP	CD117 ADC	Mice	None	3–5	> 99% Host HSC depletion 98% Donor myeloid chimerism > 99% BM HSC donor chimerism	[56]
CD117 Saporin + anti-CD4, CD8, CD40L MAbs	CD117 ADC	Mice	None	15	High and sustained donor chimerism in 14/15 mice Tolerance to skin allograft	[57]
CD117-SAP	CD117 ADC	Mice	Haemophilia A	6	Robust depletion of HSCs 90.6% Donor myeloid chimerism at 4 weeks	[58]
MGTA-117	CD117 ADC	Mice	AML	3	> 95% Host HSPC depletion Dual benefit as conditioning and anti-tumour treatment	[61]
DCR-2-PBD	CD300f ADC	Mice	AML	5	97% Reduction in total CD34 + cells Selective depletion of myeloid cells	[72]
CD45-SAP or CD117-SAP	45 and 117 ADCs	Mice	FA	16	Significant depletion of HSCs Improved engraftment versus cytarabine-conditioned group	[73]
CD45-SAP + CD117-SAP + Baricitinib	45 and 117 ADCs	Mice	None	35	Significant depletion of HSCs 99% Donor myeloid chimerism	[74]
Iomab-B	CD45 RlAb	Phase 3	AML	153	Trial ongoing: preliminary results: 99% Depletion of circulating blasts 91% of patients > 95% donor chimerism	[79–82]
90Y-BC8	CD45 RlAb	Phase 1	AML, CML, MDS, ALL, RA	15	87% complete remission All engrafted by day 28 2 yr OS 46%	[83, 84]
90Y-BC8	CD45 RlAb	Phase 1	Plasma Cell Myeloma	15	0% TRM 100% Donor chimerism of CD3 and CD33 cells 5 yr OS/PFS = 71%/41%	[85]
90Y-BC8	CD45 RlAb	Phase 1	B-NHL, T-NHL, HL	21	0% Day 100 NRM Median day 13 neutrophil and platelet engraftment 5 yr OS/PFS = 68%/37%	[86]
131I-BC8	CD45 RlAb	Phase 2	AML, MDS	15	Completed, no results	[87]
211A-BC8-B10	CD45 RlAb	Phase	Non-Malignant Neoplasms	40 (Estimated)	Recruiting	[88]
211A-BC8-B10	CD45 RlAb	Phase 1/2	AML, ALL, MDS, AL, CML	50 (Estimated)	Recruiting	[89]
90Y-Daclizumab	CD25 RlAb	Phase 1/2	HL	4	100% CR ongoing 4.5–7 yr	[102]
90Y-Anti-CD25	CD25 RlAb	Phase 2	HL	33 (Estimated)	Recruiting	[103]
90Y-Basiliximab	CD25 RlAb	Phase 1	NHL	20 (Estimated)	Ongoing	[104]
90Y-Anti-CD66	CD66 RlAb	Phase 2	AML, ALL, MDS, immuno-deficiency, anaemia	30	93% Stable engraftment 43% Malignant disease relapse, 6% non-malignant relapse 37% aGvHD, 17% cGvHD 94% 2 yr OS non-malignant group 69% 2 yr OS malignant group	[108]
90Y-Anti-CD66	CD66 RlAb	Phase 1	Paediatric leukaemia	9	Completed, no results	[109]
90Y-Anti-CD66	CD66 RlAb	Phase 1/2	Leukaemia, myeloma, lymphoma	62	Completed, no results	[110]
90Y-Anti-CD66	CD66 RlAb	Phase 2	Paediatric leukaemia	25 (Estimated)	Active, not yet recruiting	[111]
Anti-CD7 CAR with CXCR4 receptor	Anti CD7-CAR-T	Mice	None	5	27% LT-HSC donor chimerism 20–30% PB granulocyte, B and T cell donor chimerism	[113]
Anti-CD117 CAR-T*	Anti-CD7 CAR-T	Mice	None	10	98% CD117 + cell elimination Reduction in bone marrow cellularity	[114]
Anti-CD123- CAR-T*	Anti-CD123-CAR-T	Mice	AML	31	Eradication of normal haematopoiesis in CD34 + cell transplanted mice	[115]
Anti-CD123- CAR-T*	Anti-CD123-CAR-T	Mice	None	-	Reduced CD34 + cell clonogenic capacity Impaired self-haematopoietic system reconstitution	[116]
CD34-CD3 BiTE*	CD34-CD3 BiTE	Mice	None	5	Reduced BM and splenic tumour burden, HSC depletion	[118]
FLT3-CD3 BiTe	FLT3-CD3 BiTe	Mice	AML	5	Increased PD-1 expression on T cells Decreased PB leukaemic burden Modest survival advantage compared to PD-1 treatment	[120]

Summary of antibody-based conditioning therapies reported in this review. (*) indicates potential use in conditioning; however, the main goal of the study was not to examine conditioning potential

*ADC*: antibody–drug conjugate, *aGvHD*: acute graft-versus-host disease, *AL*: acute leukaemia, *ALL*: acute lymphocytic leukaemia, *AML*: acute myeloid leukaemia, *BM*: bone marrow, *B-NHL*: B cell Non-Hodgkin lymphoma, *cGvHD*: chronic graft-versus-host disease, *CML*: chronic myeloid leukaemia, *CR*: complete remission, *FA*: Fanconi anaemia, *HL*: Hodgkin lymphoma, *HSC*: haematopoietic stem cell, *HSPC*: haematopoietic stem and progenitor cells, *LT-HSC*: long-term haematopoietic stem cell, *MAb*: monoclonal antibody, *MDS*: myelodysplastic syndrome, *NRM*: non-relapse mortality, *OS*: overall survival, *PB*: peripheral blood, *PFS*: progression-free survival, *RA*: refractory anaemia, *SCID*: severe combined immunodeficiency, *TBI*: total body irradiation, *T-NHL*: T cell non-Hodgkin lymphoma, *TRM*: transplant-related mortality, *TTE*: time to engraftment

## Monoclonal/naked antibodies

### Alemtuzumab

Alemtuzumab (CAMPATH-1H) is an anti-CD52 humanised IgG1 antibody developed in 1988 [17]. CD52, also known as CAMPATH-1 antigen, is expressed by many immune cells, including lymphocytes, monocytes, macrophages and dendritic cells [18]. Alemtuzumab is US Food and Drug Administration (FDA)-approved for the treatment of B cell chronic lymphocytic leukaemia (B-CLL) [19] and multiple sclerosis [20] and is being explored as a lymphodepleting agent in conditioning regimens, primarily to reduce the incidence of GvHD.

Use of alemtuzumab within conditioning regimens is associated with good survival outcomes in malignant and non-malignant disease settings. This is particularly prominent in paediatric studies, where patient cohorts with a range of malignant and non-malignant disease, including primary immunodeficiencies, various leukaemia types and MDS, treated with alemtuzumab in conjunction with classic conditioning regimens of fludarabine and treosulfan or busulfan, exhibited > 77% 3-year overall survival (OS) [21–24]. In these instances, rates of event-free survival (EFS) were also substantial, with a 1-year post-HCT EFS of 61% in patients treated for haematological malignancies [23]. In patients treated for primary immunodeficiency, EFS was longer, with 78% experiencing EFS 5 years post-HCT [21]. Long-term survival benefits have also been shown, with 66% of lymphoma patients alive 10 years post-transplant, of whom 45% were progression-free [25]. Other studies have shown conflicting results, with alemtuzumab-based conditioning conferring no survival benefit in adult acute myeloid leukaemia (AML) patients, although cGvHD incidence was lower. Prognosis was far poorer for relapsed/refractory lymphoma patients, although this could be attributed to changes within broader clinical practice [26–28]. Poor outcomes associated with alemtuzumab-based conditioning could also be attributed to alemtuzumab-induced T cell depletion, reducing the graft-versus-leukaemia (GvL) effect [27], and the fact that the patients treated with alemtuzumab in these instances are already high-risk, defined by having previously failed high-dose therapy and auto-HCT [28].

Inclusion of alemtuzumab improved engraftment outcome in certain studies. Sustained donor chimerism was achieved in almost all patients [22, 23]. Further studies revealed mixed chimerism in both the myeloid and T cell lineages, albeit to a greater extent in the T cell lineage. Importantly, mixed chimerism was not associated with increased risk of key outcome measures, such as OS, GvHD and relapse [25]. Donor lymphocyte infusions (DLI) were necessary to achieve full chimerism in most cases [25, 26]. Indeed, a greater number of DLI have been required in patients whose conditioning regimens contained alemtuzumab, although this could be as a result of disease relapse [26].

HCT in alemtuzumab-conditioned patients was generally well tolerated, with a low (15%) incidence of grade III/IV acute or chronic GvHD (aGvHD, cGvHD) in paediatric studies [21–23, 28]. Adults experienced a lower rate of cGvHD; just 3% of patients with a range of haematological malignancies who underwent a BEAM–alemtuzumab or fludarabine–melphalan–alemtuzumab conditioning regimen suffered cGvHD. However, 51% of patients in this study exhibited aGvHD [29]. As expected, transplants from an unrelated donor present substantial risk of GvHD. Incidence of cGvHD seems to be higher in certain diseases, such as AML, where inclusion of alemtuzumab in the conditioning regimen reduced overall cGvHD incidence from 70 to 23%. Furthermore, extensive cGvHD reduced from 47 to 4% [26]. Importantly, age appears unrelated to increased cGvHD incidence when alemtuzumab is included in the conditioning regimen [29], perhaps providing an option for older patients, for whom RIC may be more beneficial.

Nevertheless, the relationship between GvHD and GvL is finely balanced. In a study of 201 adult patients with haematological malignancies (mostly AML and NHL), patients received allo-HCT following conditioning with alemtuzumab incorporated into either BEAM or fludarabine and melphalan. Survival data showed that patients with grades II–IV and grades III–IV had an increased risk of death (HR 1.64 and 2.83). However, GvHD could also be a protective factor. During the follow-up period, of those that died without showing GvHD (*n* = 49), 69.4% of deaths were due to relapse, whereas this figure dropped to 25% in those that died after aGvHD (*n* = 32) and 30% after cGvHD (*n* = 10) [29], indicating a protective effect of GvHD against relapse, although patients who experienced aGvHD grades II–IV had poorer survival (grade I not reported). This highlights the balance needed in reducing GvHD deaths while also ensuring enough GvHD to cause GvL, thus perhaps altering the alemtuzumab dosage or schedule may be beneficial.

Studies have shown a high risk of bacterial, fungal and viral infections after conditioning containing alemtuzumab, due to the induced immunosuppression [22, 26, 28]. Furthermore, Epstein–Barr virus (EBV) reactivation can occur due to the depletion of memory T cells that normally control the disease in its latent state within B cells. Indeed, one study found that in 111 allo-HCT patients with haematological malignancies, all of whom were conditioned with a regimen containing alemtuzumab, the 2-year cumulative reactivation of EBV was 40.3% [30]. Treatment with rituximab successfully reduced the viral load in these patients to normal levels.

Ultimately, alemtuzumab-mediated depletion of lymphocytes can be effective in reducing GvHD. However, such immunosuppression brings its own risks, including infection, reduced GvL and disease progression. Thus, inclusion of alemtuzumab is likely only suitable in a subset of patients, although it has shown promise in elderly patients, for whom optimal conditioning regimens can be more difficult to determine.

### Rituximab

The anti-CD20 monoclonal antibody rituximab is approved for the treatment of many diseases, including non-Hodgkin lymphoma (NHL), chronic lymphocytic leukaemia (CLL) and rheumatoid arthritis [31]. Notably, the approval of rituximab in NHL was the first FDA-approved antibody-based therapy in the cancer setting. The CD20 receptor is expressed largely on B cells; thus, rituximab promotes B cell depleting mediated immunosuppression.

Understanding the contribution of rituximab to conditioning regimens is complicated by its success as a front-line drug. In many studies comparing rituximab vs non-rituximab cohorts, the non-rituximab cohort has already received rituximab as therapy. Only 9% of adult allo-HCT patients in the CIBMTR registry received rituximab in their conditioning regimen, and in the trials investigating BEAM versus rituximab–BEAM conditioning, 76% of the non-rituximab group had rituximab exposure immediately before HCT [32, 33]. No difference was seen in survival or relapse outcomes [32, 33]. Addition of rituximab to these conditioning regimens did not impact aGvHD incidence, or time-to-occurrence [33, 34]. These studies suggest that addition of rituximab to conditioning regimens for B cell malignancies may not be beneficial. However, some studies involved a wide range of diseases, which were not evenly distributed between the rituximab and non-rituximab groups, with AML most represented in the non-rituximab cohort, and lymphoma most represented in the rituximab cohort. Importantly, there were no AML patients in the rituximab cohort [34]. Therefore, these findings could be due to different disease responses to rituximab, which should be explored further.

Addition of rituximab to conditioning regimens led to a high PFS/low relapse rate in B cell NHL, CLL and follicular lymphoma [35–37]. In a study concerning B cell malignancies, in which the non-rituximab patients had never received rituximab as part of a treatment plan, cGvHD incidence was halved, and OS increased from 54 to 72% in the rituximab-conditioned cohort [38]. Importantly, there was no difference in relapse incidence, suggesting that the immune suppression does not hinder anti-tumour responses.

Ultimately, the benefits of using rituximab in pre-transplant conditioning regimens remain unclear; while there are promising results seen in trials and retrospective data analysis, its excellence as a first-line therapy may supersede benefits seen in transplant conditioning. More work is needed to understand the usefulness of rituximab in conditioning regimens alone.

### Vedolizumab

Vedolizumab is a humanised anti-α4β7 integrin MAb, which is FDA-approved for the treatment of ulcerative colitis and Crohn’s disease [39]. In the HCT setting, vedolizumab has been incorporated into a conditioning regimen to reduce lower-intestinal aGvHD through blocking the migration of gut-homing T lymphocytes via the α4β7 integrin receptor. The intestinal tract is a major site of aGvHD, representing the majority of morbidity/mortality, and is key in amplifying systemic aGvHD [40]. α4β7 is a key mediator in inflammation; therefore, blocking the receptor may prevent aGvHD [40]. In the HCT setting, it has been trialled as lower-intestinal aGvHD prophylaxis in a phase 1b study of 24 patients with either AML, ALL or MDS with disease state in remission. Patients received either a MAC or RIC regimen with low-dose (*n* = 3, 75 mg) or high-dose (*n* = 21, 300 mg) vedolizumab, administered on days -1, + 13 and + 42. The study was encouraging, with 0% and 19% of patients experiencing grade II–IV aGvHD by day 100 in the low- and high-dose groups, respectively. Patient outcomes were good, with 1 of the 3 low-dose and 2 of the 21 high-dose patients experiencing relapse. This correlated with survival, with 12-month survival of 84.7% in the high dose and 66.6% in the low dose, although the low patient number may skew results [40]. Following this study, a phase 3 trial is currently investigating vedolizumab at the 300 mg dose as intestinal aGvHD prophylaxis in conditioning in patients with a haematological malignancy or myeloproliferative disorder [41].

### Antibodies in Trials

Monoclonal antibodies targeting CD117 are a growing area of research, with pre-clinical antibodies such as JSP191 [42], FSI-174 [43] and ACK2 [44] having been developed. CD117 is the receptor for SCF, a critical cytokine for HSC survival, maintenance and proliferation in the HSC niche [9]. Therefore, blocking the receptor deprives HSCs of SCF signalling, leading to HSC depletion.

Pre-clinical in vivo work has shown promise of JSP191 (formerly AMG191). Bone marrow HSPCs were depleted in mouse xenograft models and immunocompetent cynomolgus macaques, after 12 weeks of JSP191 treatment [42, 45]. Such depletion is key for successful engraftment. Interestingly, when they gave a further HSC transplant to mimic the allo-HCT process in mice, donor chimerism was observed vs unconditioned mice, thus showing JSP191 permitted engraftment in humanised NSG mice [45]. Of the CD117 monoclonal antibodies, JSP191 has advanced the furthest, with phase I/II in-human clinical trials investigating the addition of JSP191 in conditioning regimens for AML and MDS [46], severe combined immunodeficiency (SCID) [47] and Fanconi anaemia [48].

Early results from the JSP191 SCID trial show that the drug is well tolerated, with 4/6 patients at > 24 weeks post-HCT showing successful engraftment (> 5% donor granulocyte chimerism). Furthermore, all have shown the production of donor-derived T or B cells after 36 weeks [49].

Elsewhere, FSI-174 depleted BM HSCs when combined with magrolimab (anti-CD47 Ab) in non-human primates, although this was not seen when administered as a single agent [43]. Additionally, no other cytopenias occurred, suggesting a high specificity of this combination of antibodies.

A 2016 study using the anti-mouse-CD117 antibody ACK-2 in combination with an engineered anti-CD47 antibody [50] demonstrated a requirement for administration of both antibodies to impact HSC survival and ultimately eliminated > 99% HSCs in immunocompetent mice. Considerable CMP and GMP depletion was also observed, causing substantial BM HSPC microenvironment clearance, which facilitated effective donor HSC engraftment.

Additional antibody combinations have been investigated. Notably, a six-antibody cocktail containing anti-CD117, anti-CD47, anti-CD4, anti-CD8, anti-CD40L and anti-CD122 has been investigated in mice [51]. Anti-CD117 and anti-CD47 depleted HSCs by blocking survival and anti-phagocytic signalling, allowing macrophage-mediated depletion. Anti-CD122 eliminated NK cells, anti-CD40L and anti-CD4 acted against T-helper cells, and anti-CD8 against cytotoxic T cells. In all immunocompetent mice tested (*n* = 5), conditioning with the 6-antibody method enabled efficient engraftment of donor HSCs, showing high donor chimerism after 8 weeks. Importantly, donor cells were fully MHC-mismatched, suggesting that this strategy could overcome this major hurdle to successful HCT.

## Antibody–drug conjugates (ADCs)

ADCs combine a toxic payload to an antibody by a short linker molecule. As a result, they combine the toxicity of classic chemotherapy with the specificity of antibody-mediated cell targeting (reviewed extensively in [52]).

### CD45

An anti-CD45-saporin ADC was investigated in an immunocompetent mice model of sickle cell anaemia, in which treatment and HCT led to complete correction of disease [53]. Saporin is a toxin that inactivates ribosomes but lacks a cell entry mechanism; therefore, conjugating to an antibody allows entry while minimising the risk to non-target expressing cells [54]. Results were impressive, with a donor 4-month BM HSC chimerism of 90% and donor peripheral blood chimerism of 75–90%. Furthermore, there was a 99% depletion of host stem cells. Compared to traditional TBI, there were equal levels of engraftment (measured by chimerism), yet toxicity was considerably reduced, measured by quicker B and T cell recovery, preserved anti-fungal immunity, bone marrow structural integrity and avoided neutropenia and anaemia [53].

CD45-saporin was also investigated in SCID mouse models [55]. Following anti-CD45-saporin treatment, 95.2% and 91.7% HSC depletion was observed in immunodeficient *RAG1-KO* and *RAG1-mutant* mice, respectively, as well as 85.9% and 76.3% depletion of LSK cells (HSCs and progenitors). Interestingly, synergism was observed when anti-CD45-saporin and 2 Gy of TBI were combined (94.7% and 100% HSC depletion) representing a possible area for future research. This combination also effectively induced high levels of donor chimerism, allowed rapid immune reconstitution and resulted in high multilineage engraftment.

### CD117

Anti-CD117-saporin ADCs have also been studied. Czechowicz et al. developed the ADC using the 2B8 anti-mouse-CD117 MAb clone [56]. In immunocompetent WT mice, administration of the ADC led to a > 99% depletion of HSCs, allowing effective engraftment, evidenced by 98% donor myeloid chimerism in peripheral blood 4 weeks post-HCT. Furthermore, there were high levels of T and B cell donor chimerism 20 weeks post-transplant. BM analysis revealed > 99% BM HSCs were donor and were successfully re-transplanted to secondary recipients, confirming HSC function. Importantly, no neutropenia and lymphopenia were observed, preserving immunity when assessed by challenging with LCMV virus and *Candida albicans* fungus. Furthermore, humanised mice studies using saporin conjugated to an anti-human-CD117 (104D2 clone) indicated marked human HSC depletion in vivo. These data show the potential for ADC-mediated HSC depletion in conditioning.

In immunocompetent mice with induced immunosuppression using anti-CD4, anti-CD40L and anti-CD8 antibodies, conditioning with the aforementioned anti-CD117-saporin enabled full MHC mismatch transplantation, observed by high donor chimerism almost 2 years post-transplant [57]. Impressively, anti-CD117-saporin conditioning also led to immune tolerance to skin allografts. In unconditioned and isotype control saporin-conditioned mice, skin allografts were rejected due to the MHC mismatch. However, in anti-CD117-saporin-conditioned mice, 13 of the 15 mice accepted skin grafts and experienced long-term survival, showing an induced state of tolerance, although there was immune cell infiltration into the allograft site. This has added potential to allow patients without MHC-matched donors to potentially be eligible for transplant.

An anti-CD117-saporin ADC was examined in a haemophilia A mouse model [58]. While progenitor cell depletion was not observed 5 days after treatment, HSCs were markedly reduced. Similarly to previous studies, 4-week post-HCT donor myeloid chimerism was high at 91%, increasing to 95% after 16 weeks. Combination of the ADC with an antibody-mediated immunosuppression approach, consisting of anti-CD4, anti-CD8 and anti-CD40L antibodies, proved effective when transplanting lentiviral-modified donor HSPCs. All mice conditioned in this way produced effective multilineage donor chimerism 30 weeks post-HCT (myeloid = 65%, T cell = 88%, B cell = 82%), without any cases of immune rejection. Transplantation of the modified HSPCs seemed to correct for haemophilia A, evidenced by the large reduction in bleeding in the mice receiving gene therapy.

Anti-CD117 has also been conjugated to amanitin, an RNA polymerase II inhibitor [59–61]. Amanitin is very hydrophilic, meaning that it does not readily enter cells. In fact, native amanitin has a 20,000-fold decreased cytotoxicity versus an antibody-conjugated form, rendering it extremely useful as an ADC payload due to an in-built safety feature [62]. Amanitin is also effective against dormant cells, meaning that it can target HSCs [62].

The earliest study examining a panel of payloads conjugated to an anti-CD117 antibody revealed amanitin as the only payload able to deplete human HSCs in humanised NSG mice by > 90% [59]. This was improved in cynomolgus monkeys, with > 95% HSC depletion observed 7 days post-treatment with a single dose of anti-CD117-amanitin. Importantly, white blood cell and lymphocyte levels remained stable for the duration of the 8-week study, indicating that this approach spares the adaptive immune system. Further studies explored the use in rhesus macaque monkeys, with HCT performed after ADC treatment. Again, ADC administration was effective and well tolerated, resulting in > 99% host HSPC depletion while preserving lymphocytes. Donor cells engrafted successfully, with donor-derived neutrophils, platelets and granulocytes detectable after 10 days [60].

The antibody was further developed by optimising the linker molecule and engineering a short half-life of 91 h in NSG mice. This ADC came to be known as MGTA-117 and exhibited > 95% depletion of host HSPCs in humanised mice [61]. MGTA-117 demonstrated anti-leukaemic activity, providing dual benefit as both conditioning and anti-cancer treatment. In humanised NSG models mimicking treatment naïve and refractory AML, the ADC consistently showed an extended survival versus cytarabine treatment [61].

### CD33

Gemtuzumab ozogamicin (GO) is one of the most well-studied ADCs—an anti-CD33 antibody conjugated to the anti-tumour antibiotic calicheamicin. GO is currently approved for the treatment of CD33^+^ AML, both newly diagnosed and relapsed/refractory [63]. The CD33 antigen is widely expressed in cells of the myeloid lineage, including early progenitors and mature cells [64]. Similar to rituximab mentioned previously, the influence of GO in conditioning regimens is difficult to ascertain due to its frequent use as an AML therapy.

Initial GO studies suggested inferior survival outcomes, with 46% of patients who received GO prior to HCT in AML and MDS alive after 100 days, compared to 81% of patients who had not [65]. This was attributed to toxicity, and an association between GO and hepatic veno-occlusive disease (VOD) was identified when GO was administered within 3.5 months before intensive conditioning and HCT [65]. Of 62 patients, 13 (21%) developed VOD, although 69% of these had previously received high-dose (6 or 9 mg/m^2^) GO. Subsequent studies revealed lower dose (2 mg/m^2^) GO was tolerable and could be safely added to a RIC regimen, with just 1 out of 44 patients experiencing reversible VOD, 82% showing treatment response at the lower dose and median OS was 11 months [66]. Another phase I/II trial of 31 patients investigated the use of a 6 and 3 mg/m^2^ dosing strategy 3 and 2 weeks prior to HCT, still equalling a 9 mg/m^2^ cumulative dose, followed by RIC. This strategy was effective, with all 24 evaluable patients showing engraftment, and only one case of hepatic SOS. However, outcome measures remained relatively low, 24-month relapse incidence was 38% and estimated 24-month OS and EFS being 39% and 35%, respectively [67].

More recently, follow-up of the phase 3 ALFA-0701 clinical trial [68], which investigated the addition of GO to standard chemotherapy in the initial treatment of adult de novo AML, demonstrated that GO did not increase risk of VOD/SOS relative to the control arm after post-therapy HCT. Interestingly, no differences were observed in post-transplant survival outcomes, although GO did improve OS in patients not receiving HCT. The lack of outcome difference may be due to 97% of patients who received GO ≥ 2 months before HCT [69]. Nevertheless, the study indicated that GO was indeed safe for use prior to HCT.

In contrast, higher doses (up to 7.5 mg/m^2^) were found to be very effective and tolerable in children and adolescents with poor-risk CD33 + AML, when GO was added to a busulfan/cyclophosphamide conditioning regimen before allo-HCT [70]. Of the 12 patients, day 100 TRM was 0% and engraftment was successful, with the patients achieving a median 30-day donor chimerism of 99%. The results from this study meant this conditioning regimen has progressed and is currently being investigated in a phase II trial in high-risk CD33 + AML and MDS patients [71].

### CD300f

A novel target for conditioning could be CD300f. The receptor was expressed more than CD33, the target of GO, in AML blasts, and was expressed by HSCs and progenitors [72]. The ADC showed synergism with fludarabine, an agent used in many conditioning regimens, in cytotoxicity of AML cell lines, further indicating its potential for inclusion in RIC. In vivo studies of AML xenografted mice showed that the ADC significantly depleted bone marrow HSPCs and peripheral blood myeloid cells, leading to ADC-treated mice showing an extended survival versus untreated and isotype control. Lymphoid cells were spared as these cells do not express CD300f, which may impact the risk of rejection in a HCT model [72]. Nevertheless, CD300f seems a promising target, and further research on HCT following CD300f-ADC conditioning is needed.

### Combination ADCs

Administration of CD45-saporin and CD117-saporin as single agents was beneficial in a mouse model of Fanconi anaemia [73]. HSCs and progenitors were effectively depleted and resulted in engraftment, as both conditions resulted in > 10% chimerism 12 weeks post-HCT. Importantly, engrafted HSCs showed multilineage potential, with donor-derived myeloid and lymphoid cells present in peripheral blood after 12 weeks, and by 24 weeks there was no difference in total lymphocytes, granulocytes and monocytes versus untreated mice. Compared to mice conditioned with cyclophosphamide—an established conditioning agent, all ADC-treated mice showed similar or superior engraftment by 8 weeks, highlighting the potential of this regimen. Ultimately, treatment resulted in correction of Fanconi anaemia in mice. ADC-treated mice showed significantly reduced toxicity versus cyclophosphamide-treated mice, such as the absence of hepatic inflammation present after cyclophosphamide conditioning. Furthermore, there was no difference in weight loss between ADC-conditioned and untreated mice.

Combination of ADCs with other types of therapies, such as tyrosine kinase inhibitors, may also be an effective strategy [74]. One study combined the Janus kinase 1/2 inhibitor baricitinib with CD45 and CD117-ADCs as pre-allo-HCT conditioning in mice. Baricitinib provided immunosuppression through T and NK cell depletion. Post-HCT, donor chimerism in the myeloid lineage reached 99%, even in fully MHC-mismatched mice, and enabled stable multilineage engraftment. Notably, those treated with the ADC/baricitinib combination effectively balanced GvHD with GvL. This strategy is promising, as enabling fully MHC-mismatched transplants would expand the available donor pool for patients.

## Radio-labelled antibodies

Radiotherapy has historically been the backbone of myeloablative conditioning regimens since the 1950s, in the form of total body irradiation (TBI), reviewed extensively elsewhere [75]. TBI undoubtedly improves transplant success rate (such as increased 5 year leukaemia-free survival post-HCT, compared to busulfan/cyclophosphamide conditioning) by effectively depleting BM cellularity, eradicating malignant cells and inducing immunosuppression to reduce the risk of transplant rejection [76]. Nevertheless, extensive and severe toxicities are associated with TBI, including VOD, stomatitis, interstitial pneumonitis and neurologic complications, amongst others [75].

Many TBI side effects result from the systemic administration of radiotherapy; therefore, a targeted delivery system of radiation to the BM could maintain the efficacy while reducing toxicities. To this end, radio-labelled antibodies have been trialled as part of conditioning regimens.

### CD45

The CD45 receptor is expressed on almost all haematopoietic cells, besides mature erythrocytes [77, 78]. Radio-labelled antibodies against CD45 have been widely studied, with many trials currently undergoing.

The phase III SIERRA trial is investigating Iomab-B, an ^131^Iodine-anti-CD45 antibody as part of a conditioning regimen including fludarabine and low-dose TBI, for relapsed/refractory AML [79]. Preliminary results [80–82] show that dosing with Iomab-B as a single agent significantly depleted circulating blasts by 99% [81]. All of the Iomab-B patients engrafted with a median time to neutrophil engraftment of 14 days, with 91% showing full donor chimerism (> 95%) by day 100. Notably, 78% of patients randomised to the conventional care, non-Iomab-B group failed therapy. As a result, 49% crossed over to the Iomab-B group, with engraftment results consistent with patients from the original Iomab-B group. The results indicated that targeted radiation to the BM allowed effective engraftment in patients with a heavy leukaemia burden [82].

The ^90^Yttrium-BC8 anti-CD45 antibody added to a conditioning regimen containing fludarabine and low-dose TBI before allo-HCT has been investigated in a phase I clinical trial in high-risk AML, ALL and MDS [83]. Of the 15 participants, 13 (87%) showed complete remission and all engrafted by day 28. However, 6 patients relapsed, 5 of whom subsequently died from their disease, resulting in only 46% achieving 2-year OS, although it should be noted that these are high-risk patients who frequently had active disease or measurable residual disease before conditioning began [84]. Therefore, the study presented the feasibility of this conditioning regimen in these patients.

The same regimen has also undergone a phase I clinical trial in high-risk multiple myeloma [85]. In a small, dose escalation study, treatment was well tolerated, with 0% treatment-related mortality (TRM) at day 100. By day 28, all patients had 100% donor chimerism in CD3 and CD33 cells. Five-year OS and PFS were 71% and 41%, respectively, highlighting the feasibility and efficacy of this regimen in these patients.

Promising results were also seen when this antibody was added to BEAM conditioning prior to auto-HCT in phase I trials of patients with relapsed B-NHL, T-NHL, or HL. Approximately half of the patients enrolled had chemo-refractory disease [86]. Again, this was successful, with neutrophil and platelet engraftment at median day 13. Notably, 2 patients who received ^90^Y-BC8 alone had neutrophil engraftment at days 10 and 12 and platelet engraftment at days 13 and 15, showing that ^90^Y-BC8 alone permits engraftment. Five-year OS and PFS estimates were 68% and 37%. As in the aforementioned leukaemia studies, ^90^Y-BC8 was relatively well tolerated, with 0% NRM at day 100, although 76% of the 21 enrolled patients experienced at least one grade ≥ 3 non-infusion-related adverse event.

Further clinical trials examining anti-CD45 radio-labelled antibodies currently ongoing include: ^131^Iodine-BC8 with fludarabine, TBI and allo-HCT in advanced AML and MDS patients ([87], phase II, complete no results posted); ^211^Astatine-BC8-B10 plus fludarabine, cyclophosphamide and TBI before HCT in non-malignant diseases ([88], phase I/II, recruiting); and ^211^Astatine-BC8-B10 with fludarabine and TBI prior to HCT in high-risk AML, ALL, MDS or mixed-phenotype acute leukaemia ([89], phase I/II, recruiting).

### CD20

^90^Yttrium ibritumomab tiuxetan (Zevalin), an anti-CD20 antibody conjugated to the radioactive isotope ^90^Yttrium, was the first radioimmunotherapy drug approved for cancer treatment, when it was FDA-approved in 2002 for the treatment of NHL [90]. More recently, however, trials have investigated its use in conditioning regimens as a cytoreductive agent.

A matched cohort analysis of 92 diffuse large B cell lymphoma (DLBCL) patients receiving either TBI or Z-BEAM (Ibritumomab tiuxetan plus BCNU, etoposide, cytarabine and melphalan) before HCT found that Z-BEAM had improved outcomes vs TBI. This included improved 4-year OS (81% vs 52.7%) and non-relapse mortality (0% vs 15.8%). Z-BEAM was better tolerated, with significantly fewer grade ≥ 3 long-term toxicities (39% vs 70%), and 100-day mortality was 0% vs 8.7%. Overall, the study found that Z-BEAM vs TBI improved transplant outcomes and reduced toxicity incidence [91].

Consistent with Ibritumomab tiuxetan being effective in higher-risk patients, a study of Z-BEAM vs BEAM alone in refractory/relapsed aggressive lymphoma showed improved outcomes in the Z-BEAM arm. All patients showed engraftment after HCT. Intermediate-risk patients had improved PFS in Z-BEAM vs BEAM (69% vs 29%), and 2-year OS of all patients was 91% vs 62% [96], showing a large improvement in patient outcome with Z-BEAM.

Similarly, impressive results were seen by another group using the Z-BEAM regimen in a multicentre trial in diffuse large B cell lymphoma (DLBCL) [97], a disease associated with poor prognosis after the transformation from indolent NHL to DLBCL. All patients receiving HCT engrafted; outcomes following Z-BEAM conditioning and HCT were very encouraging—2-year OS was 90% and PFS was 68%, although 9 patients died prior to HCT. Toxicity was relatively low, with a single grade 3 pulmonary toxicity, although there were 14 incidences of grade 3/4 infections among the 54 patients in the trial.

A trial in mantle cell lymphoma comparing rituximab–BEAM (R-BEAM) (*n* = 35) vs Z-BEAM (*n* = 11) found a trend but no significant difference in 5-year OS (55% vs 71%), and no difference in 4-year PFS (32% vs 41%), toxicities or engraftment, concluding that both antibody-containing regimens are well tolerated [98].

A phase II trial also combined Ibritumomab tiuxetan with low-dose TBI and fludarabine as conditioning in high-risk (chemo-resistant, bulky or aggressive) B cell NHL [92]. Ibritumomab tiuxetan was used to provide disease control and early cytoreduction. Forty high-risk patients were enrolled, of which 24 (60%) showed early responses to conditioning treatment. Impressively, 59% of chemo-resistant patients showed response. Following HCT, 30-month OS, PFS and NRM were 54%, 31% and 16%, respectively. Importantly, all patients showed sustained engraftment, with 28-day donor chimerism in CD3 and CD33 PB cells being 96% and 100%. Mild to moderate aGvHD was observed in 27 patients, and 4 experienced grade 3 aGvHD [93]. Longer-term follow-up of this trial was examined for disease outcome and toxicity [94]. At a median follow-up of 9 years, 27.5% of patients were alive and NRM was 30%, with deaths attributed to infection, GvHD and pneumonia. Regarding cGvHD, 11 patients experienced chronic extensive GvHD, although no complications directly attributed to Ibritumomab tiuxetan were observed. The regimen was most effective in patients with indolent disease, yet DLBCL patients fared poorly. Overall, the addition of Ibritumomab tiuxetan was safe, well tolerated, induced early response and prolonged survival in high-risk B cell NHL patients, particularly those with indolent histologies.

Indeed, another study of indolent B cell NHL found that patients that received Ibritumomab tiuxetan in their conditioning regimens did better than those without. Ibritumomab tiuxetan was more frequently given to patients with high-risk, chemo-resistant disease, but after adjusting for disease state, Ibritumomab tiuxetan-treated patients had 3-year PFS and OS of 71% and 87%, respectively, vs 44% and 59% in those without [95].

A recent single-arm phase II clinical trial adding Ibritumomab tiuxetan to low-intensity chemotherapy (rituximab, bendamustine and fludarabine) prior to allo-HCT was investigated in lymphoma [99]. Of the 20 patients enrolled, 2 suffered treatment-related mortality and 14 were alive after 3 years (70% OS), representing an encouraging transplant outcome. However, 60% suffered from serious adverse events, primarily febrile neutropenia (40%), infection and infestation (40%).

### CD25 (IL-2Rα)

To date, studies examining radio-labelled CD25 antibodies are an emerging topic; thus far, only pre-clinical and early-phase clinical trial studies have been reported. CD25 is highly expressed on activated CD4 + or CD8 + effector T cells, T regulatory cells, as well as macrophages and dendritic cells [100, 101].

A small (*n* = 4) phase I/II trial was carried out for the anti-CD25 antibody daclizumab conjugated to ^90^Yttrium, as an addition to a BEAM conditioning regimen in relapsed/refractory Hodgkin lymphoma [102]. All 4 patients registered were heavily pre-treated before the regimen began. The auto-HCT treatment was effective in all 4 patients, as they all experienced complete responses ongoing after 4.5–7 years post-transplant. Ongoing clinical trials of CD25 antibodies include ^90^Yttrium–basiliximab plus BEAM prior to auto-HCT in HL ([103], phase II, recruiting) and ^90^Yttrium–basiliximab plus BEAM prior to auto-HCT in T cell NHL ([104], phase I, active).

### CD66

CD66 is part of the carcinoembryonic antigen (CEA) family of receptors, which are expressed on various myeloid and lymphoid cells, such as granulocytes and B cells, as well as in malignant disease, such as NHL and MM [105–107]. ^90^Yttrium-anti-CD66 radio-labelled antibodies have been successfully added to conditioning regimens in a phase II trial of 30 paediatric and adolescent patients with advanced disease, primarily AML, ALL, MDS and immunodeficiencies, as well as anaemias [108]. Due to the targeted nature of the antibody, in all but 3 patients the BM absorbed at least twice the dose of radiation compared to other organs except the spleen, and effective BM cell depletion was observed. All but 2 patients achieved stable engraftment, and all surviving patients achieved normal blood counts. Low toxicity was observed, and 2-year OS was 83%, although this was skewed in favour of patients with non-malignant transplant indications (94%) vs malignant (69%). Overall, the regimen was safe and showed the feasibility for use in young people.

Other clinical trials for CD66 radio-antibodies include ^90^Yttrium-anti-CD66 as part of RIC in childhood relapsed/refractory leukaemia ([109], phase I, complete no results posted), a phase I/II trial in leukaemia, myeloma and lymphoma of ^90^Yttrium-anti-CD66 added to conditioning ([110], complete no results posted), and ^90^Yttrium-anti-CD66 as part of RIC in childhood high-risk leukaemia ([111], phase II, active not yet recruiting).

## T cell therapies

Chimeric antigen receptor T (CAR-T) cells are an emerging immunotherapy involving a genetically modified T cell, whereby the CAR is an engineered synthetic receptor designed to target a specific antigen [112].

In vivo studies of a CD117 CAR-T cell showed the potential of CAR-Ts in conditioning. Firstly, in vitro experiments showed effective killing of CD117 + HSCs and HSPCs from mice. Further in vivo work showed a significant reduction in BM CD117 + cells, long-term HSCs (LT-HSCs) and progenitor cells by day 8. In a mouse HCT model, cyclophosphamide conditioning alone did not allow engraftment, but in CAR-T plus cyclophosphamide mice, average donor chimerism of LT-HSCs was 26.9% (*n* = 5), and by 12 weeks there was significant donor chimerism of around 20–30% of PB B cells, T cells and granulocytes. Lastly, in a chronic granulomatous disease mouse model, a conditioning regimen of CAR-T with cyclophosphamide followed by HCT allowed phenotypic correction of the disease [113].

Another anti-CD117 CAR-T cell induced specific killing of CD117 + AML cell lines and healthy HSPCs killing in vitro and in vivo [114]. In fact, 98% of CD117 + cells were eliminated by CAR-T cells, resulting in a marked reduction in overall BM cellularity [114]. While this study was not performed in the HCT conditioning setting, the depletion of healthy HSPCs and AML cells shows promise as a dual benefit in conditioning.

Another target could be CD123. The α chain of the IL3 receptor is frequently expressed in AML blasts and normal haematopoietic cells [115]. One group engineered a CD123 CAR-T and showed potent killing of human AML cell lines and primary cells in mouse xenograft models [115]. Also, the treatment eradicated normal haematopoiesis in mice transplanted with healthy human CD34 + cells [115].

Another anti-CD123 CAR-T effectively eliminated CD123 + AML cell lines in vitro and in vivo [116]. In vitro studies showed that CAR-T treatment massively reduced the clonogenic ability of primary CD34 + HSPCs. In vivo*,* mice were irradiated and human CD34 + HSPCs were transplanted. Administration of the CAR-T after either 1 day or 6 weeks significantly impaired the ability of the cells to engraft and for multilineage reconstitution [116]. While the authors remarked that this should be a sign for caution therapeutically, the results show a potential as conditioning therapy or as a pre-conditioning treatment, to reduce tumour burden while also depleting the marrow.

Bispecific T cell engagers (BiTEs) allow the targeting of two antigens simultaneously, which can bring together two different cell types. Most commonly, BiTEs are composed of two linked artificial antibodies targeting a tumour antigen and CD3, a T cell antigen, in order to redirect and engage T cells against a tumour cell [117].

A CD34-CD3 BiTE has recently been developed for prospective use in the AML setting, although results showed potential for use in conditioning. Ex vivo HSCs isolated from PBSC grafts were significantly depleted after co-culturing with T cells and the BiTE. In vivo studies using CD34 + AML cell lines transplanted into NSG mice again showed significant reductions in tumour burden in the spleen and BM. Although not intended or examined for use in conditioning, the elimination of HSCs and elimination of CD34 + BM cells in vivo represents a potential for examination in conditioning [118].

Kiefer et al. developed a CD117-CD3 BiTE capable of effectively killing CD117 + AML cell lines and primary AML blasts in vitro*.* Notably, CD117 + primary HSPCs were also killed, demonstrating an effective way to deplete HSPCs in vitro [119].

The FLT3 receptor is normally restricted to HSCs and HSPCs to promote differentiation, but is expressed on AML blasts in a majority of patients where it confers blast survival and proliferation. Therefore, the receptor represents a target for AML treatment, as well as potentially for a conditioning agent. One group developed and tested a FLT3-CD3 BiTE in a humanised AML mouse model. They observed effective elimination of xenografted AML cell lines and healthy human haematopoietic cells in the BM [120]. This result showed a potential use of a FLT3-CD3 BiTE in both eliminating disease and effective conditioning.

## Discussion

Immunotherapy is an exciting area of research that is hugely important in how we treat disease. Technologies such as CAR-T cells and bispecific antibodies are still in their infancy; while the first monoclonal antibody, muromonab-CD3, was FDA-approved in 1986, the first CAR-T approval, tisagenlecleucel, was as recent as 2017. This may explain why fewer CAR-T therapies have been tested in conditioning versus antibodies. Questions remain over CAR-T cells’ safety profile, including the concept of administering ‘living drugs’, prompting investigation of suicide genes and on/off mechanisms [121]. Once these concerns have been addressed, we may see a greater number of these newer immunotherapy types tested in conditioning.

One of the biggest issues regarding HCT is the conditioning toxicity, limiting patients’ eligibility for this potentially curative treatment. Cancer is often a disease of the elderly, yet ≥ 65 years has classically been used as a cut-off for HCT eligibility, although more emphasis is now being placed on physiological age rather than actual age, using predictors such as the HCT comorbidity index [122–124]. Currently, considerations are often made based on the planned regimen, such as using RIC in older patients [124]. Though it is difficult to ascertain the exact figure of ineligible patients due to the complex criteria of assessing patient eligibility [124], creating less toxic conditioning regimens using immunotherapy could broaden patients’ eligibility for HCT, therefore increasing probabilities of survival.

Infection following HCT remains a major problem. In a study of paediatric HCT patients, admission rates to intensive care units because of infection were as high as 45.7%, with 22.2% mortality [125]. This is due to the systemic immunosuppression required to prevent graft rejection and GvHD, and the conditioning regimen influences the risk of serious infection. Myeloablative regimens prolong neutropenia and lymphopenia, while reduced intensity regimens require a higher dose of immunosuppressive drugs. Thus, both heighten the risk of infection [126]. Also, the choice of GvHD prophylaxis plays a major role in post-transplant immunosuppression. Indeed, studies have shown a high risk of bacterial, fungal and viral infections after conditioning containing alemtuzumab [22, 26, 28]. Therefore, lymphodepleting immunotherapy agents should be considered, such as alemtuzumab, ATG and rituximab which deplete T and B cells, respectively.

One concern for using a less toxic conditioning regimen may be an increase in disease relapse. Some studies found a correlation between RIC and higher relapse vs MAC regimens [127]. Notably, however, the reduction in transplant-related mortality (TRM) in RIC meant overall survival was not significantly different between the groups [127]; therefore, it is often a balancing act between reducing TRM while managing relapse risk. Increased relapse may be explained by reduced GvL owing to the similarity between GvL and GvHD, as well as the presence of residual malignant cells that would have been eliminated by a more intense regimen. These factors present a considerable challenge when considering an all-immunotherapy regimen, as current trials target markers of healthy cell types. Utilising GvL is important as it is the main anti-malignancy mechanism in RIC transplants.

Efforts made to separate and enhance GvL using immunotherapy methods have been explored, such as ‘designing’ grafts whereby the number and ratio of cytotoxic T cells, regulatory T cells, NK cells and γ δ T cells are carefully adjusted before infusion to the patient, or adoptive transfer of these cells and more (such as CAR-T and dendritic cells) to induce GvL [128]. Inclusion of an anti-cancer immunotherapy drug either before or during the conditioning regimen may reduce relapse incidence, as seen by the inclusion of rituximab in conditioning for patients with B cell malignancies. Similarly, addition of a targeted inhibitor, such as the Janus kinase 1/2 inhibitor baricitinib, can be included with antibody-based conditioning to help balance GvHD and GvL [74]. Furthermore, labelled antibodies, such as ADCs, may prevent relapse through the bystander effect. This occurs when the ADC payload is released from the target cell after internalisation and degradation of the complex, leading to neighbouring cells, such as malignant blasts, taking up the payload and being killed [129]. This effect may lead to malignant cells being eliminated while not being directly targeted.

Nevertheless, immunotherapy is not without its challenges. For instance, infusion reactions, cytokine release syndrome/cytokine storm (commonly seen with alemtuzumab and rituximab), immunogenicity and autoimmunity are well-characterised side effects of immunotherapy and therefore need to be monitored when used in conditioning [121, 130]. However, the shorter treatment time for conditioning (typically between 1–2 weeks before transplant), compared to immunotherapy as a disease treatment, may circumvent these issues that are seen in longer-term treatment strategies.

Clinical trials thus far have used immunotherapy with, rather than instead of, a standard conditioning regimen, typically only with a single immunotherapy agent. Therefore, the true potential of immunotherapy-only conditioning may be being hidden, as the other agents may be causing toxicity or other negative effects. A multi-agent, immunotherapy-only regimen may be an option in the future, as a 6-antibody cocktail was effective as conditioning in mice models [51]. While such studies in humans are not currently feasible, this approach may reveal that combining multiple immunotherapy drugs that target different cell types may eliminate the need for chemotherapy or radiotherapy, while providing better results.

The HSC immunophenotype is complex and not fully understood, so identifying novel markers, such as CD35 or CD11a [131], may present new targets. Furthermore, known HSC markers, such as CD90 [132], could be examined for targeting by new immunotherapy agents to provide more comprehensive and accurate bone marrow depletion.

Antibody-based conditioning regimens may be the next big advancement in HCT. By removing the toxicity associated with traditional conditioning methods, more patients could be eligible to receive this potentially curative treatment. In this way, transplant outcomes could be improved, such as TRM and GvHD, ultimately improving patient survival.

## Abbreviations

ADC: Antibody–drug conjugate
aGVHD: Acute graft-versus-host disease
ALL: Acute lymphoblastic leukaemia
AML: Acute myeloid leukaemia
B-CLL: B cell chronic lymphocytic leukaemia
BiTE: Bispecific T cell engagers
BM: Bone marrow
CAR-T: Chimeric antigen receptor T cells
CD: Cluster differentiation
cGvHD: Chronic graft-versus-host disease
CMP: Common myeloid progenitor
DLBCL: Diffuse large B cell lymphoma
DLI: Donor lymphocyte infusion
EBV: Epstein–Barr virus
EFS: Event-free survival
FDA: Food and Drug Administration
GMP: Granulocyte–monocyte progenitor
GvHD: Graft-versus-host disease
GVL: Graft versus leukaemia
HLA: Human leukocyte antigen
HSC: Haematopoietic stem cell
HCT: Haematopoietic stem cell transplantation
HSPC: Hematopoietic stem and progenitor cells
MAC: Myeloablative conditioning
MDS: Myelodysplastic syndrome
MM: Multiple myeloma
NHL: Non-Hodgkin lymphoma
NK: Natural killer
NRM: Non-relapse mortality
OS: Overall survival
PB: Peripheral blood
PFS: Progression-free survival
RIC: Reduced intensity conditioning
SCF: Stem cell factor
SCID: Severe combined immunodeficiency
SOS: Sinusoidal obstructive syndrome
TBI: Total body irradiation
TRM: Transplant-related mortality
VOD: Veno-occlusive disease
WHO: World Health Organisation
